# Direct measurement of ferroelectric polarization in a tunable semimetal

**DOI:** 10.1038/s41467-021-25587-3

**Published:** 2021-09-06

**Authors:** Sergio C. de la Barrera, Qingrui Cao, Yang Gao, Yuan Gao, Vineetha S. Bheemarasetty, Jiaqiang Yan, David G. Mandrus, Wenguang Zhu, Di Xiao, Benjamin M. Hunt

**Affiliations:** 1 Department of Physics, Carnegie Mellon University, Pittsburgh, PA USA; 2 International Center for Quantum Design of Functional Materials (ICQD), Hefei National Laboratory for Physical Sciences at the Microscale, University of Science and Technology of China, Hefei, Anhui China; 3 Materials Science and Technology Division, Oak Ridge National Laboratory, Oak Ridge, TN USA; 4 Department of Materials Science and Engineering, University of Tennessee, Knoxville, TN USA; 5 Department of Physics and Astronomy, University of Tennessee, Knoxville, TN USA

**Keywords:** Two-dimensional materials, Characterization and analytical techniques, Ferroelectrics and multiferroics

## Abstract

Ferroelectricity, the electrostatic counterpart to ferromagnetism, has long been thought to be incompatible with metallicity due to screening of electric dipoles and external electric fields by itinerant charges. Recent measurements, however, demonstrated signatures of ferroelectric switching in the electrical conductance of bilayers and trilayers of WTe_2_, a semimetallic transition metal dichalcogenide with broken inversion symmetry. An especially promising aspect of this system is that the density of electrons and holes can be continuously tuned by an external gate voltage. This degree of freedom enables measurement of the spontaneous polarization as free carriers are added to the system. Here we employ capacitive sensing in dual-gated mesoscopic devices of bilayer WTe_2_ to directly measure the spontaneous polarization in the metallic state and quantify the effect of free carriers on the polarization in the conduction and valence bands, separately. We compare our results to a low-energy model for the electronic bands and identify the layer-polarized states that contribute to transport and polarization simultaneously. Bilayer WTe_2_ is thus shown to be a fully tunable ferroelectric metal and an ideal platform for exploring polar ordering, ferroelectric transitions, and applications in the presence of free carriers.

## Abstract (web summary)

Free carriers and electrical polarization coexist in ferroelectric metals. Here, the authors use a capacitive method to probe the electronic compressibility of free carriers in a tunable semimetal, extract the polarized contribution, and study the carrier dependence of the ferroelectric state.

## Introduction

Polar materials exhibit charge separation in the absence of an applied electric field, an effect of broken inversion symmetry and a unique polar axis in the crystal^1,2^. In certain polar systems, the charge polarization can be switched by an external electric field, an effect known as ferroelectricity. In principle, the presence or absence of ferroelectric effects depends only on the crystal class and not on the details of the electronic structure. Despite this, nearly all known conventional ferroelectrics are electrically insulating. Since the first theoretical proposals for ferroelectric metals in 1965^1^, only a handful of experimental claims of ferroelectric-like phases in metallic systems have been reported^3–9^. Many such claims fail to demonstrate two key signatures of ferroelectric behavior—direct evidence of the polarization and ferroelectric switching—due to bulk screening effects. With reports of polar switching in the metallic state of WTe_2_^10–12^, a strong case for a ferroelectric metal has emerged.

**Fig. 1:**
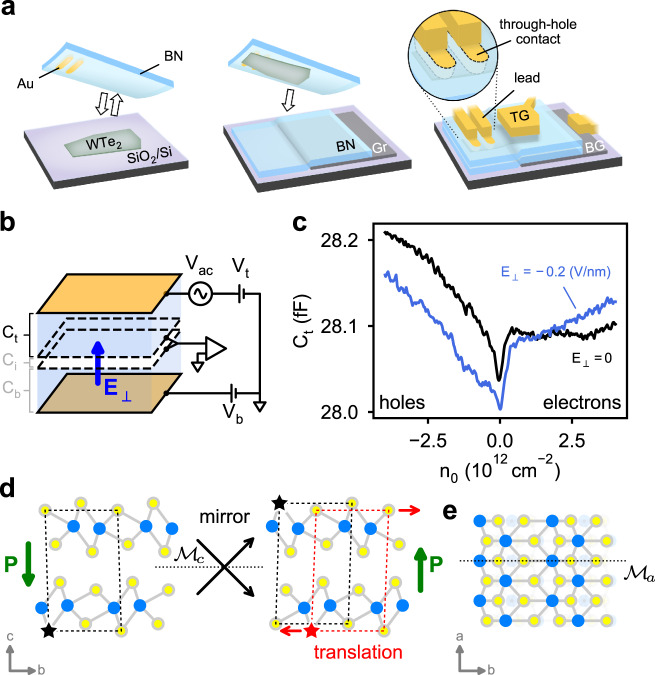
Fabrication and measurement of the bilayer capacitance device. **a** Schematic of our lithography-free encapsulation and contact method, using a boron nitride (BN) crystal previously prepared with through-hole Au contacts to pick up and transfer WTe_2_ on to another BN dielectric layer with a graphite bottom gate below. Top gate and leads to the through-hole contacts and bottom gate are patterned after fully encapsulating the WTe_2_. **b** Measurement schematic showing the measurable capacitances: *C*_t_, between the top gate and WTe_2_, and *C*_b_, between the bottom gate and WTe_2_. *C*_*i*_ is the interlayer capacitance across the bilayer. **c** Measured top capacitance, *C*_t_, as a function of carrier density, *n*_0_, at zero and finite electric field, *E*_⊥_. **d** Side-view structure of bilayer WTe_2_ in two stable configurations, each showing the net polarization state along *c*-axis (**P**). The two states are related equivalently by a mirror operation along the *c*-axis (${{{{{{{{\mathcal{M}}}}}}}}}_{c}$) or by lateral translation between the layers along the *b*-axis of 0.72 Å^18,19^, or ~11% of the unit cell. The ⋆ symbol labels a Te atom before and after the mirror operation (black) and translation (red) as a visual guide. The mirror/translation equivalence allows a subtle shift between the layers to switch the structure between polarization states. **e** Top-view structure showing the only invariant symmetry of the crystal, mirror reflection along the *a*-axis (${{{{{{{{\mathcal{M}}}}}}}}}_{a}$).

Here we focus on the polar, semimetallic van der Waals (vdW) crystal, T_d_-WTe_2_, in the limit of two atomic layers, thin enough to admit an external electric field (Fig. 1d, e). Few-layer crystals of WTe_2_ have drawn recent interest for exhibiting a wide variety of low-temperature phases^13–17^. Recent transport measurements showed that bilayer and trilayer WTe_2_ exhibit intrinsic, switchable electrical polarization in the conducting state by detecting the out-of-plane polarization with a monolayer graphene sensor^10^. Separately, surfaces of bulk WTe_2_ crystals were shown to display hysteresis in piezoresponse force microscopy^11^. Subsequent first-principles calculations indicated that the net polarization points only in the out-of-plane direction and that the underlying mechanism results from a subtle interlayer sliding between the layers in two stable configurations^18,19^, an effect later demonstrated by second harmonic generation^12^. The in-plane rigidity of the lattice adds an unfavorable energy cost to the formation of neighboring dipoles with opposite polarization, facilitating long-range order and leading to macroscopic polarization. Combined with low carrier density and a thickness less than the out-of-plane screening length^11,20^, these factors conspire to stabilize a ferroelectric metal state in bilayer WTe_2_. These findings are exciting given the semimetallic and tunable nature of bilayer WTe_2_, which enables reaching both electron and hole bands by electrostatic gating in the ferroelectric state. While the detection of polarization and hysteresis in previous experiments^10,11^ is promising, a measurement of the metallic polarization as a function of charge density for electrons and holes in WTe_2_ is still missing. In particular, using a remote graphene layer previously enabled direct detection of the out-of-plane polarization^10^ but prevented independent control of the charge density and electric field. Crucially, this limitation precluded observing the effect of free carriers on the polarization and its dependence on carrier type and density, a fundamental open question for ferroelectric metals.

In this work, we directly measure the charge polarization and electronic compressibility as a function of density for electrons and holes with independent control of the electric field. We study the simplest polar WTe_2_ system, a bilayer, via capacitive sensing in a dual-gated, planar capacitance device (Fig. 1b). Capacitance measures the electronic compressibility (and thus metallicity) of a two-dimensional (2D) system. In a bilayer 2D system, the top- and bottom-gate capacitances provide a direct measurement of the *layer-specific* charge distribution^21^, and thus the out-of-plane polarization, as previously demonstrated in bilayer graphene^22^. Furthermore, the parallel-plate geometry enables this charge sensing with simultaneous and independent control of the vertical electric field and the carrier density in the bilayer by electrostatic gating.

## Results

Our devices each consist of a bilayer WTe_2_ crystal encapsulated by two hexagonal boron nitride (hBN) dielectric layers, with metallic top and bottom gates, and contacts integrated into the top hBN layer^23^ (Fig. 1a).

We measure the capacitance between the top gate and the bilayer, *C*_t_, while applying DC voltages to the top and bottom gates to tune the (nominal) total carrier density, ${n}_{0}\propto {C}_{t}^{0}{V}_{t}+{C}_{b}^{0}{V}_{b}$, and out-of-plane electric field, ${E}_{\perp }\propto {C}_{t}^{0}{V}_{t}-{C}_{b}^{0}{V}_{b}$ (Fig. 1b). While the geometric contributions to the capacitance (${C}_{t}^{0}$ and ${C}_{b}^{0}$) are constant, there are additional contributions to the measured capacitance *C*_t_ from the electronic compressibility. In a bilayer system with partial electric field penetration, the layer-specific densities *n*_*i*_ can differ between the two layers (*i* = 1, 2) for a given total density *n* = *n*_1_ + *n*_2_, particularly in the presence of an electric field. The electrostatic potentials of each layer *ϕ*_*i*_ depend on the top- and bottom-gate voltages and, crucially, any built-in electric field in the bilayer^21,22^. As such, *ϕ*_*i*_ in each layer can also differ, even in thermodynamic equilibrium. The electronic compressibility of a 2D bilayer is generally described by a 2 × 2 matrix, *ν*_*ij*_ = − ∂*n*_*i*_/∂*ϕ*_*j*_; however, in a weakly coupled bilayer it is possible to characterize the system with only the diagonal elements, *ν*_*ii*_, the layer-specific compressibilities. Due to the vdW nature of the interlayer coupling, in bilayer WTe_2_ this is indeed the case (Supplementary Fig. 9). Subsequently, the top capacitance may be written,

1 $${C}_{{{{{{\rm{t}}}}}}}\approx {C}_{{{{{{\rm{t}}}}}}}^{0}\left(1-\frac{{C}_{{{{{{\rm{t}}}}}}}^{0}}{{e}^{2}{\nu }_{11}}\right),$$

(see “Methods” for details). The second term in Eq. (1) is a quantum correction to the geometric capacitance ${C}_{{{{{{\rm{t}}}}}}}^{0}$, inversely proportional to the layer-specific compressibility of the top layer, *ν*_11_. The layer-specific densities *n*_1_ and *n*_2_ can be obtained by integrating the capacitance, and thus the polarization, proportional to *p* = *n*_1_ − *n*_2_, can be measured.

**Fig. 2:**
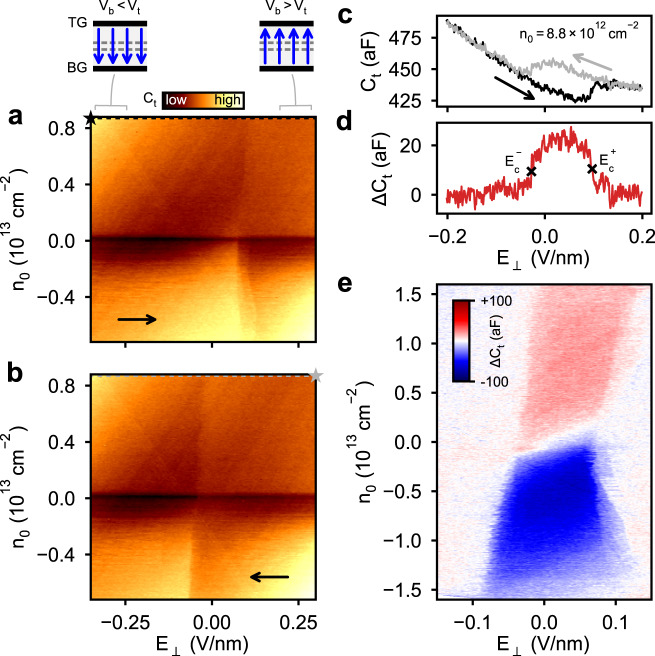
Hysteresis in the electric field response for electrons and holes. **a** Forward and **b** backward scans of the top capacitance *C*_t_ as a function of electric field, *E*_⊥_, for a range of carrier densities, *n*_0_. **c** Capacitance traces measured along the dashed lines in **a**, **b** (beginning at the ⋆ symbol in each case) displaying a smooth background as well as sudden jumps at electric field values that depend on the sweep direction. **d** Difference between the traces in **c**. **e** Compilation of differences between forward and backward scans in **a**, **b** for an extended range of carrier densities, showing the change in sign of the switching from electrons to holes and the gradual density dependence of the switching behavior.

For fixed, external field *E*_⊥_ = 0, the measured *C*_t_ as a function of the electron density exhibits a minimum near charge neutrality, *n*_0_ ≈ 0 (Fig. 1c). The minimum in *C*_t_ indicates the presence of a small band gap (incompressible state) in the WTe_2_ bilayer, a feature consistent with previous observations of a sharp drop in the conductance of bilayer WTe_2_ in transport^10,24^. At fixed, negative electric field, the capacitance minimum becomes more prominent, suggesting that the gap is electric field tunable (see Supplementary Fig. 4), similar to bilayer graphene^22,25^. However, in contrast to bilayer graphene the electric field response is robustly asymmetric around *E*_⊥_ = 0, as shown in the full *n*_0_ and *E*_⊥_ dependence in Fig. 2a. This effect results from the absence of an inversion center or mirror plane between the layers of bilayer WTe_2_, implying the crystal is polar along the *c*-axis (Fig. 1e).

To probe the switching behavior of the polar direction, we sweep the electric field back and forth at fixed density, as shown in Fig. 2a, b. Sudden changes are observed in *C*_t_ at all densities: *C*_t_ jumps at a positive critical electric field value ${E}_{{{{{{\rm{c}}}}}}}^{+}$ when sweeping toward positive *E*_⊥_ (Fig. 2a), whereas the critical field ${E}_{{{{{{\rm{c}}}}}}}^{-}$ is negative when sweeping in the negative direction (Fig. 2b), forming a hysteresis loop (Fig. 2c) at each density. Taking the difference of two representative sweeps in opposite directions ${{\Delta }}{C}_{{{{{{\rm{t}}}}}}}\equiv {C}_{{{{{{\rm{t}}}}}}}^{\leftarrow }-{C}_{{{{{{\rm{t}}}}}}}^{\to }$ (Fig. 2d), we see that *C*_t_ overlaps nearly everywhere excluding the hysteretic region between the critical fields, ${E}_{{{{{{\rm{c}}}}}}}^{\pm }$, where *C*_t_ is multi-valued. The critical fields generally fall within ∣*E*_⊥_∣ ≲ 0.1 V/nm and appear to be weakly dependent on charge density (Fig. 2e). Interestingly, the sign of the hysteretic difference switches for holes (Δ*C*_t_ < 0) compared to electrons (Δ*C*_t_ > 0), and the magnitude of the difference decreases at large densities of either sign.

**Fig. 3:**
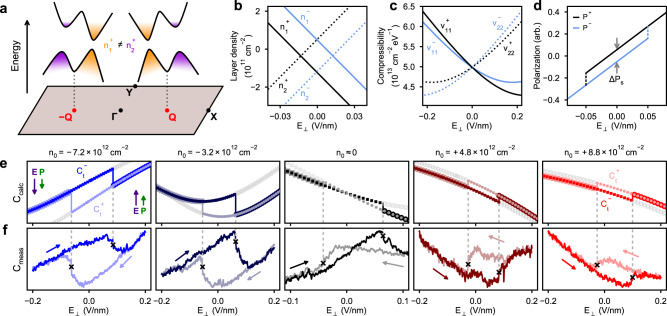
Layer-polarized bands yield distinct capacitance branches for each polarization state. **a** Schematic low-energy bands showing layer-polarized valleys (orange and purple) in the *P*^+^ state, for small electron density and *E*_⊥_ = 0. In the *P*^−^ state, the colors and layer polarization would be interchanged. Each pair of valleys is centered around a point along Γ–*X*, labeled *Q*. Band parameters are exaggerated to emphasize separation of valleys. **b** Representative calculated layer densities, **c** compressibilities, and **d** polarization $\propto {n}_{1}^{\pm }-{n}_{2}^{\pm }$ in each polarization state, ±, from *n*_0_ ≈ 0. **e** Computed top capacitances, ${C}_{{{{{{\rm{t}}}}}}}^{+}$ and ${C}_{{{{{{\rm{t}}}}}}}^{-}$ in the *P*^+^ and *P*^−^ states, respectively, for the listed densities versus electric field, with colored symbols indicating the hysteretic path observed in experiment, while gray symbols denote portions of each capacitance branch that are inaccessible in experiment due to switching behavior (switching fields are not computed in the model; solid vertical lines are shown extending from experimental switching fields in **f** to reflect the loop observed in the experiment). Capacitance is calculated using computed layer densities, potentials, and compressibilities to evaluate Eq. (11a). **f** Measured top capacitance hysteresis loops for matching electron and hole densities in **e**.

Switching behavior is clearly present in the capacitance, but to understand how this relates to the polarization we must first recognize that the ground state structure of bilayer WTe_2_ possesses two stable configurations with opposite polarization^12,18,19^ (Fig. 1d). Similarly, from Fig. 2a–c, we see that there are two stable values for *C*_t_ in the central region between the two critical fields, ${E}_{{{{{{\rm{c}}}}}}}^{\pm }$. To reveal how the *C*_t_ bistability relates to the two polarization states, we employ a self-consistent calculation of the capacitance using a **k** ⋅ **p** model for the bilayer WTe_2_ bands^26^ together with an exact form of Eq. (1) (see Eq. (11a) in “Methods”). The **k** ⋅ **p** Hamiltonian (Eq. (4)) provides the low-energy electronic structure, shown schematically in Fig. 3a, and layer-projected wavefunctions in each polarization state. The layer-specific densities *n*_*i*_ (Fig. 3b) and electric potentials *ϕ*_*i*_ are obtained from the bands by performing a self-consistent electrostatics calculation for the parallel-plate system (see “Methods”). The compressibilities (Fig. 3c) are obtained by numerical differentiation and inserted into the general form of Eq. (1) to compute the capacitance (given by Eq. (11a)). Since there are two stable configurations for the bilayer, these quantities all depend on the polarization state, yielding different values ${n}_{i}^{\pm }$, ${\phi }_{i}^{\pm }$, and ${\nu }_{ii}^{\pm }$ in each case. Using these calculated quantities, the polarization at fixed *n*_0_ can be determined from the difference ${P}^{\pm }=e{d}_{i}({n}_{1}^{\pm }-{n}_{2}^{\pm })$ for interlayer separation *d*_*i*_. Evaluating this directly, we can construct a schematic polarization loop that illustrates both the dielectric polarization response as well as the spontaneous polarization from the computed layer densities (shown for *n*_0_ ≈ 0 in Fig. 3d, with switching behavior added manually for illustration). The difference between the layer imbalance in the two polarization states yields the change in the spontaneous polarization, Δ*P*_s_ ≡ *P*^+^ − *P*^−^, as indicated for *E*_⊥_ = 0 in Fig. 3d.

**Fig. 4:**
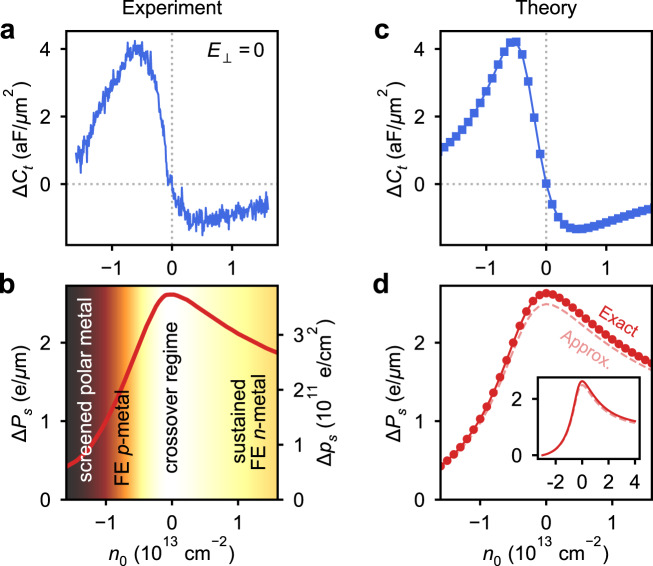
Ferroelectric polarization in the presence of free carriers. **a** Measured density dependence of ${{\Delta }}{C}_{{{{{{\rm{t}}}}}}}\equiv {C}_{{{{{{\rm{t}}}}}}}^{+}-{C}_{{{{{{\rm{t}}}}}}}^{-}$ at *E*_⊥_ = 0. **b** Change in measured spontaneous polarization calculated by integration, Δ*P*_s_ ∝ ∫Δ*C*_t_d*n*_0_. Left axis provides 2D polarization units while the right scale is given in terms of charge separation between the layers, Δ*p*_s_ = Δ*P*_s_/*d*_*i*_ for WTe_2_ interlayer separation, *d*_*i*_ = 0.7 nm. Background shading follows the magnitude of Δ*P*_s_, illustrating distinct regions of ferroelectric (FE) behavior. **c** Computed Δ*C*_t_ and **d** Δ*P*_s_ based on model Hamiltonian for bilayer WTe_2_, with inset in **d** showing computed Δ*P*_s_ for an extended density range (with identical units). Δ*P*_s_ is computed by two different methods: “Exact” using Eq. (18), and “Approx.” using Eq. (33).

Polarization switching reflects a reversal of the density imbalance between the layers (Fig. 3a, b) and manifests as switching in the top-gate capacitance due to the inequivalence of the layer-specific compressibility in the two polarization states (Fig. 3c). For instance, in Fig. 3e, we show calculated curves for *C*_t_ as a function of *E*_⊥_ at a few selected electron and hole densities using the calculated compressibilities. The result of the calculation is two capacitance curves, ${C}_{{{{{{\rm{t}}}}}}}^{+}$ and ${C}_{{{{{{\rm{t}}}}}}}^{-}$, that correspond to the polarization states *P*^+^ and *P*^−^, respectively. Comparing to the experiment (Fig. 3f), for sufficiently large positive (negative) *E*_⊥_, we only measure the ${C}_{{{{{{\rm{t}}}}}}}^{+}$ (${C}_{{{{{{\rm{t}}}}}}}^{-}$) branch. In the hysteretic region, the polarization state *P*^+^ or *P*^−^ depends on the direction of the electric field sweep, and thus the measured capacitance jumps between the ${C}_{{{{{{\rm{t}}}}}}}^{+}$ and ${C}_{{{{{{\rm{t}}}}}}}^{-}$ branches at the coercive fields, ${E}_{{{{{{\rm{c}}}}}}}^{\pm }$. While our model does not include all the details of the electronic structure (Supplementary Fig. 6), and thus we do not expect a perfect match with experiment, the general trends of the capacitance with *E*_⊥_ and *n*_0_ are captured nicely, for instance, the observation that ${C}_{{{{{{\rm{t}}}}}}}^{+} \, > \, {C}_{{{{{{\rm{t}}}}}}}^{-}$ for electrons and ${C}_{{{{{{\rm{t}}}}}}}^{+} \, < \, {C}_{{{{{{\rm{t}}}}}}}^{-}$ for holes. Next we will see that some of the details of the electronic structure are less relevant for difference quantities such as Δ*C*_t_ and Δ*P*_s_, which leads to improved agreement between the model and experiment, as shown in Fig. 4.

Finally, we address the density dependence of the polarization to ascertain the effect of adding free charge to a polarized semimetal. Previously, we defined Δ*C*_t_ as the difference between the right-sweeping and left-sweeping *C*_t_ curves. Having identified the ${C}_{{{{{{\rm{t}}}}}}}^{\pm }$ curves in the central region of each hysteresis loop, we may now equate ${{\Delta }}{C}_{t}={C}_{t}^{+}-{C}_{t}^{-}$ for ${E}_{c}^{-} \, > \,{E}_{\perp } \, > \, {E}_{c}^{+}$. Figure 4a shows the density dependence of Δ*C*_t_ for *E*_⊥_ = 0, equivalent to a vertical line cut from Fig. 2e. The non-monotonic curve follows the size of the *C*_t_ hysteresis loop, showing a maximum and minimum on either side of charge neutrality and trending toward zero at large densities. The difference Δ*C*_t_ evaluated at *E*_⊥_ = 0 is related to a *dipole compressibility*, ∂*p*/∂*n*_0_ (see “Methods”), a measure of spontaneous dipole formation as carriers are added to the system. This quantity yields the change in layer polarization density *p* = *n*_1_ − *n*_2_ as the chemical potential is varied (via the combination of gate voltages, *n*_0_) and thus is not relevant in ferroelectric insulators. By integrating Δ*C*_t_ with respect to carrier density, we obtain the change in spontaneous polarization as a function of density along *E*_⊥_ = 0,

2 $${{\Delta }}{P}_{{{{{{\rm{s}}}}}}}\propto \int {{\Delta }}{C}_{{{{{{\rm{t}}}}}}}\ {{{{{\rm{d}}}}}}{n}_{0}.$$

The result of this integration along *E*_⊥_ = 0 is shown in Fig. 4b (including geometrical prefactors given in Eq. (33)). Intuitively, the spontaneous polarization is strongest at charge neutrality, where there are few free carriers available to screen the built-in polarization. Adding electrons or holes to the system reduces the net polarization. However, a pronounced asymmetry emerges in the spontaneous polarization depending on the carrier type, with the polarization decreasing faster from its value at charge neutrality for holes compared to electrons. Thus, holes appear to screen the polarization much more effectively than electrons for equivalent charge densities. This effect can be understood by considering the layer character of the available states in the conduction and valence bands. First-principles calculations indicate that the low-energy states are intrinsically layer-polarized (see Supplementary Fig. 6), even in the absence of an external electric field. Though there are states from both layers in the conduction and valence bands, the cumulative effect of filled states up to the Fermi level results in a net polarization due to an imbalance of layer character, particularly near the band edges (see Supplementary Fig. 7). The magnitude of this imbalance is larger for valence band states compared to the conduction band. Consequently, adding holes to the valence band quickly suppresses the net spontaneous polarization, whereas adding electrons to the conduction band weakly screens it, allowing a net polarization to persist to relatively large densities (Fig. 4b). Alternatively, in an all-electron picture, adding electrons to a partially filled valence band acts to increase the net polarization due to the cumulative layer polarization of the filled states. Conduction band electrons possess an opposite layer polarization relative to the valence band and thus partially screen the cumulative polarization of the valence band as carriers are added (see Supplementary Fig. 7). Notably, the free carriers at the Fermi level are layer-polarized and thus contribute to the polarization, contrary to the simple electrostatic picture of screening in bulk metals.

## Discussion

Broken inversion and mirror-*z* symmetries together with the ultrathin, layered structure conspire to allow layer-polarized states at the Fermi level. These broken symmetries and large spin–orbit coupling further contribute to the pronounced *e*–*h* asymmetry (see Supplementary Fig. 8). This asymmetry manifests as several distinct ferroelectric regimes with both bound and free charges contributing to the net polarization. At large hole densities, the measured polarization quickly trends toward zero, entering a screened polar metal regime. In this phase, there remains underlying polarized bound charge made up of remote states; however, there are enough filled states with opposing layer character to screen this charge, resulting in a suppressed net polarization. On the other hand, at large electron densities the polarization decreases more slowly, exhibiting a persistent, *n*-type metallic ferroelectric state. Near charge neutrality, we observe a crossover between these *p*- and *n*-type metallic ferroelectric states. The crossover regime is further complicated by the electric field dependence of the conduction and valence bands, as shown in Fig. 2a, b. From *E*_⊥_ = 0, the charge neutral behavior appears to transition from a ferroelectric semimetal with finite band overlap to a ferroelectric insulator with a maximal band gap of ~3 meV from 0.3 V/nm > ∣*E*_⊥_∣ > 0.2 V/nm (Supplementary Fig. 4).

These interpretations are supported by our model calculations, with both the density dependence of calculated Δ*C*_t_ and the integrated Δ*P*_s_ matching well with experiment (Fig. 4c, d). The model also allows us to investigate the expected behavior over an extended density range, inaccessible in experiment due to dielectric breakdown of hBN. As shown in the inset of Fig. 4d, the calculated Δ*P*_s_ continues to exhibit sustained ferroelectric behavior up to electron densities of at least *n*_0_ ≈ 4 × 10^13^ cm^−2^, whereas the *p*-type ferroelectric behavior is substantially suppressed below *n*_0_ ≈ −2 × 10^13^ cm^−2^.

In conclusion, we see that bilayer WTe_2_ may be tuned from a sustained ferroelectric *n*-type metal for *n*_0_ > 10^13^ cm^−2^ to a variable-polarization ferroelectric *p*-type metal at intermediate hole densities, followed by a screened polar metal at large hole densities. In the crossover regime between the *n*- and *p*-type ferroelectric metal states, a ferroelectric insulator or semimetallic phase can be obtained depending on the electric field. The out-of-plane polarization is largest in the neighborhood of these latter phases, reaching a charge separation of ~3.7 × 10^11^ electrons/cm^2^ over 0.7 nm between the layers, equivalent to a volume polarization density of ~0.6 mC/m^2^. This maximum and the range of measured polarization magnitudes shown in Fig. 4b are comparable to the experimental value of 2 × 10^11^ e/cm^2^ measured in bilayers without independent control of the carrier density^10^ and theoretical estimates of 3.2 × 10^11^ e/cm^2^ for bilayers^18^ and 1.2 × 10^12^ e/cm^2^ for bulk crystals^11^. While these magnitudes are small compared to state-of-the-art ferroelectric insulators, the ability to tune the magnitude of the spontaneous polarization in situ and the ease of integration into vdW heterostructures is especially promising for future investigations. The ferroelectric behavior in each metallic phase is supported by the strong layer-polarized character of the states, a result of broken inversion and mirror symmetries combined with weak interlayer coupling. Together, these observations and the ingredients in WTe_2_ that lead to them provide a recipe for engineering and measuring new ferroelectric metal systems. Looking forward, we anticipate that metallic ferroelectricity will manifest in additional transition metal dichalcogenide and vdW structures^27–33^, enabling a host of new experiments in this under-explored regime.

## Methods

### Fabrication of capacitance devices

WTe_2_ is an especially air-sensitive material, quickly degrading in ambient conditions. To avoid air exposure during the fabrication process, we first integrate metal contacts into the top BN layer and then transfer this template onto the WTe_2_ in a N_2_-filled glovebox. The metal contacts are prepared ahead of time by etching holes through the BN crystal and subsequently evaporating pure Au contacts to fill these holes. We pick up the top BN along with the integrated Au contacts and use this layer to pick up the WTe_2_ crystal using standard dry transfer techniques^23^. We then pick up a bottom layer of BN to fully encapsulate the WTe_2_ and place the stack on a graphite or PdAu bottom gate, with the WTe_2_ partially overlapping the gate but with the through-hole contacts positioned as near as possible to the gated region (Fig. 1a). At this stage, the WTe_2_ is sealed from the environment on all sides, allowing us to pattern leads and a top gate using standard electron-beam lithography and metal evaporation. The top gate is designed to overlap the WTe_2_ crystal up to the edge of the bottom gate, aligning carefully to this edge. This ensures that the entire gated region of the crystal is dual-gated, preventing single-gate features in our capacitance measurements.

### Capacitance measurement

Capacitance measurements were performed by connecting the bilayer WTe_2_ in each device to a capacitance bridge circuit (Supplementary Fig. 1) with a standard, known capacitor, *C*_std_. We apply an 11 kHz alternating current (ac) excitation *δV*_t_ with an root-mean-square amplitude from 64 to 140 mV on the top gate of the device and a nearly out-of-phase signal *δV*_std_ to *C*_std_ in order to null the total ac signal at the bridge balance point. Both the amplitude and relative phase of *δV*_std_ are tuned to produce a null signal. As the total capacitance of the device changes, deviations from the null voltage are amplified by a high-electron-mobility transistor mounted within a few millimeters of the sample in the cryostat. We determine the measured top capacitance *C*_t_ from the amplified deviation from the null voltage, *C*_t_ = *C*_std_(Δ*V*_null_/*δV*_std_). All measurements were performed in a dilution refrigerator with the sample between 100 mK (as in Figs. 1, 2a–d, and 3) and 30 K (Fig. 2e) though little change was observed in the capacitance features in this range.

### **k** ⋅ **p** model for bilayer WTe_2_

To compute the layer compressibilities for bilayer WTe_2_, we adopt a **k** ⋅ **p** model for the Hamiltonian describing massive Dirac fermions in two layers with spin–orbit and interlayer coupling. Beginning with a tilted massive Dirac Hamiltonian^26^, we introduce a layer index, *i* = 1, 2, and electrostatic potential on each layer, *ϕ*_*i*_, to include the effect of a vertical electric field as well as the polarization,

3 $${\hat{{{{{{{{\mathcal{H}}}}}}}}}}_{i,s}={\phi }_{i}+t{\tilde{k}}_{x,i}+v({k}_{y}{\sigma }_{x}+\eta {\tilde{k}}_{x,i}{\sigma }_{y})+(m/2-\alpha {k}^{2}){\sigma }_{z},$$

along with a shifted *k*_*x*_-coordinate, ${\tilde{k}}_{x,i}\equiv {k}_{x}+{q}_{i}$ that accounts for the position of each layer-polarized valley (from the *Q*-point shown in Fig. 3c, for ${{{{{{{\bf{Q}}}}}}}}=Q{\hat{{{{{{{{\bf{k}}}}}}}}}}_{x}$). The Pauli matrices, *σ*_*x*,*y*,*z*_, act in the orbital pseudospin space, *s* = *↑*, *↓* is the spin degree of freedom, *η* = ±1 is a chiral index, *t* tilts the Dirac cones along *k*_*x*_, and *m* gives rise to the gap at charge neutrality (*αk*^2^ ensures convergence of ${\hat{{{{{{{{\mathcal{H}}}}}}}}}}_{i}$ as *k* → *∞*). For *E*_⊥_ = 0, the electrostatic potentials take fixed values ${\phi }_{i}({E}_{\perp }=0)={\phi }_{i}^{0}$ (see Supplementary Table 2) to account for the spontaneous polarization. The spin and layer degrees of freedom are coupled in the $\left|1,\uparrow \right\rangle$, $\left|1,\downarrow \right\rangle$, $\left|2,\uparrow \right\rangle$, $\left|2,\downarrow \right\rangle$ basis to obtain the effective Hamiltonian,

4 $${\hat{{{{{{{{\mathcal{H}}}}}}}}}}^{+}=\left(\begin{array}{cccc}{\hat{{{{{{{{\mathcal{H}}}}}}}}}}_{1\uparrow }&\hat{{{{{{{{\mathcal{P}}}}}}}}}&0&\gamma \\ \hat{{{{{{{{\mathcal{P}}}}}}}}}&{\hat{{{{{{{{\mathcal{H}}}}}}}}}}_{1\downarrow }&\gamma &0\\ 0&\gamma &{\hat{{{{{{{{\mathcal{H}}}}}}}}}}_{2\uparrow }&\hat{{{{{{{{\mathcal{P}}}}}}}}}\\ \gamma &0&\hat{{{{{{{{\mathcal{P}}}}}}}}}&{\hat{{{{{{{{\mathcal{H}}}}}}}}}}_{2\downarrow }\end{array}\right),$$

by interlayer coupling, *γ*, and spin–orbit coupling,

5 $$\hat{{{{{{{{\mathcal{P}}}}}}}}}=\left(\begin{array}{cc}{\lambda }_{x}{k}_{x}-i{\lambda }_{y}{k}_{y}&0\\ 0&-{\lambda }_{x}{k}_{x}-i{\lambda }_{y}{k}_{y}\end{array}\right),$$

where *λ*_*x*_, *λ*_*y*_ govern the spin–orbit coupling strength in the *x* and *y* directions. Such a model has previously been applied to describe the Berry curvature dipole observed in bilayer WTe_2_ in vertical electric fields^17^ and captures the spin and shifted-valley character of WTe_2_ bands determined by first-principles methods^26,34^. Here we explicitly include a polarization state label, ±, in the Hamiltonian, ${\hat{{{{{{{{\mathcal{H}}}}}}}}}}^{\pm }$, to denote the two possible configurations for the WTe_2_ bilayer. The Hamiltonian for the opposite polarization state, ${\hat{{{{{{{{\mathcal{H}}}}}}}}}}^{-}$, is obtained by interchanging layers, 1 ↔ 2, and spin. Note that both the electrostatic potential, *ϕ*_*i*_, and shifted valley coordinate, ${\tilde{k}}_{x,i}$, depend on the layer index and thus are interchanged upon a simultaneous mirror operation (${{{{{{{{\mathcal{M}}}}}}}}}_{c}$) and inversion of the electric field. As a result, the Hamiltonian possesses an identity,

6 $${\hat{{{{{{{{\mathcal{H}}}}}}}}}}^{+}({E}_{\perp })={\hat{{{{{{{{\mathcal{H}}}}}}}}}}^{-}(-{E}_{\perp }),$$

relevant for extracting the polarization from the measured capacitance, to be discussed in a subsequent section.

Though this model is single particle in nature, we note that we should view the model as describing effective single-particle orbits after electron–electron interactions are included to a certain degree. That is, screening effects due to electron–electron interactions are included in first-principles calculations at the mean field level, and our model is tuned to be consistent with the first-principles results. Furthermore, we find that the result of the model, our computed capacitance curves, match quite well with experiment, particularly when comparing to the capacitance difference in the two polarization states. As such, we conclude that additional effects of electron–electron interactions are not necessary to describe the observed polarization phenomena.

### Capacitance calculation

To understand the various features of the measured capacitance, we apply this effective model to calculate the band structure and eigenstates and numerically evaluate the capacitance for a similar geometry. Starting with Eq. (4), we determine the probability densities in each layer and sum over occupied states to obtain the layer-specific densities, *n*_1_ and *n*_2_ for the top and bottom layers, respectively. The layer-specific compressibility elements *ν*_*ij*_ are determined by taking partial derivatives of the densities *n*_*i*_ with respect to the electric potentials on each layer. The resulting compressibilities in conjunction with the geometric capacitances then determine the calculated capacitances.

The eigenstates of Eq. (4) span an 8 × 8 Hilbert space of layer, spin, and orbitals. We obtain layer densities by first calculating the probability density on each layer from the eigenstates,

7 $${\left|{\psi }_{i}({{{{{{{\bf{k}}}}}}}})\right|}^{2}=\mathop{\sum}\limits_{s,m}\left\langle ism| ism\right\rangle ,$$

for spin *s* = *↑*, *↓* and orbital pseudospin *m*. Note that here, and for the remainder of this section, we have dropped explicit labels making reference to the polarization state for simplicity. Layer densities are then determined by integration,

8 $${n}_{i}^{\prime}=\int \frac{{d}^{2}k}{{(2\pi )}^{2}}f({{{{{{{\bf{k}}}}}}}}){\left|{\psi }_{i}({{{{{{{\bf{k}}}}}}}})\right|}^{2},$$

where *f*(**p**) is the Fermi-Dirac distribution. However, Eq. (8) only includes contributions from valence electrons. The contribution from ions can be determined as follows. With the absence of an external electric field, *E*_⊥_ = 0 and ${\phi }_{i}={\phi }_{i}^{0}$, and the bilayer is at charge neutrality. We then have a vanishing total charge, ${n}_{1}^{\prime}+{n}_{2}^{\prime}+{n}_{1}^{{{{{{\rm{ion}}}}}}}+{n}_{2}^{{{{{{\rm{ion}}}}}}}=0$. On the other hand, the two layers have same ionic composition. As a result,

9 $${n}_{1}^{\,{{\mbox{ion}}}}={n}_{2}^{{{\mbox{ion}}}\,}=-\frac{1}{2}\left[{n}_{1}^{\prime}({\phi }_{1}^{0},{\phi }_{2}^{0})+{n}_{2}^{\prime}({\phi }_{1}^{0},{\phi }_{2}^{0})\right].$$

From this definition, we obtain the relevant layer densities,

10 $${n}_{i}({\phi }_{1},{\phi }_{2})={n}_{i}^{\prime}({\phi }_{1},{\phi }_{2})+{n}_{i}^{\,{{\mbox{ion}}}\,}.$$

To perform a self-consistent calculation, we treat the electrostatic potentials, *ϕ*_*i*_, as parameters and compute the layer densities, *n*_*i*_ = *n*_*i*_(*ϕ*_1_, *ϕ*_2_) for an appropriate grid of values. The compressibilities are then obtained by taking the partial derivatives *ν*_*ij*_ = ∂*n*_*i*_/∂*μ*_*j*_ = −∂*n*_*i*_/∂*ϕ*_*j*_ over the same parameter space (where *ϕ*_*i*_ = −*μ*_*i*_ for layer-specific chemical potential *μ*_*i*_ and with the sample connected to ground). Finally, the capacitances are defined in terms of small signal variations, *C*_t_ ≡ (*δn*_1_ + *δn*_2_)/*δV*_t_, *C*_b_ ≡ (*δn*_1_ + *δn*_2_)/*δV*_b_, and *C*_p_ ≡ *δn*_t_/*δV*_b_, where *C*_p_ is the penetration field capacitance, measured from the top gate to the bottom gate, for charge on the top gate *n*_t_. These expressions may be evaluated in terms of the compressibility elements (following ref. ^21^),

11a $${C}_{{{{{{\rm{t}}}}}}}={C}_{{{{{{\rm{t}}}}}}}^{0}\left[1-\frac{\,{{\mbox{det}}}\,(\hat{C})-{C}_{{{{{{\rm{b}}}}}}}^{0}{e}^{2}{\nu }_{21}+{C}_{{{{{{\rm{t}}}}}}}^{0}{e}^{2}{\nu }_{22}}{\,{{\mbox{det}}}\,({e}^{2}\hat{\nu }+\hat{C})}\right],$$

11b $${C}_{{{{{{\rm{b}}}}}}}={C}_{{{{{{\rm{b}}}}}}}^{0}\left[1-\frac{\,{{\mbox{det}}}\,(\hat{C})-{C}_{{{{{{\rm{t}}}}}}}^{0}{e}^{2}{\nu }_{12}+{C}_{{{{{{\rm{b}}}}}}}^{0}{e}^{2}{\nu }_{11}}{\,{{\mbox{det}}}\,({e}^{2}\hat{\nu }+\hat{C})}\right],$$

11c $${C}_{{{{{{\rm{p}}}}}}}={C}_{{{{{{\rm{b}}}}}}}^{0}{C}_{{{{{{\rm{t}}}}}}}^{0}\frac{{C}_{i}-{e}^{2}{\nu }_{12}}{\,{{\mbox{det}}}\,({e}^{2}\hat{\nu }+\hat{C})},$$

with geometric capacitances ${C}_{{{{{{\rm{t}}}}}}}^{0}$, ${C}_{{{{{{\rm{b}}}}}}}^{0}$, and interlayer capacitance *C*_*i*_, as shown in Fig. 1b, each given by *C*_*j*_ = *ϵϵ*_0_*A*/*d*_*j*_ using *ϵ* = 3.2 for hBN and *ϵ* = 1 within the bilayer (modeling the bilayer in the out-of-plane direction as two compressible metal sheets separated by vacuum, an assumption that does not significantly affect the outcome). The compressibility matrix, $\hat{\nu }$, is a symmetric matrix, *ν*_*ij*_ = *ν*_*ji*_, and takes the form

12 $$\hat{\nu }=\left(\begin{array}{cc}{\nu }_{11}&{\nu }_{12}\\ {\nu }_{21}&{\nu }_{22}\\ \end{array}\right),$$

while $\hat{C}$ is a matrix of geometric capacitances,

13 $$\hat{C}=\left(\begin{array}{cc}{C}_{i}+{C}_{{{{{{\rm{t}}}}}}}^{0}&-{C}_{i}\\ -{C}_{i}&{C}_{i}+{C}_{{{{{{\rm{b}}}}}}}^{0}\end{array}\right).$$

To obtain results in terms of *E*_⊥_ and *n*_0_, we first apply a transformation derived from the charge balance equations,

14a $$e{n}_{1}={C}_{{{{{{\rm{t}}}}}}}^{0}({V}_{{{{{{\rm{t}}}}}}}+{\phi }_{1}/e)+{C}_{i}({\phi }_{1}-{\phi }_{2})/e$$

14b $$e{n}_{2}={C}_{{{{{{\rm{b}}}}}}}^{0}({V}_{{{{{{\rm{b}}}}}}}+{\phi }_{2}/e)+{C}_{i}({\phi }_{2}-{\phi }_{1})/e$$

to determine *V*_b_ and *V*_t_,

15 $$\left(\begin{array}{c}{C}_{{{{{{\rm{t}}}}}}}^{0}{V}_{{{{{{\rm{t}}}}}}}\\ {C}_{{{{{{\rm{b}}}}}}}^{0}{V}_{{{{{{\rm{b}}}}}}}\end{array}\right)=e\left(\begin{array}{c}{n}_{1}\\ {n}_{2}\end{array}\right)-\hat{C}\left(\begin{array}{c}{\phi }_{1}/e\\ {\phi }_{2}/e\end{array}\right),$$

and then calculate *E*_⊥_ = (*V*_t_/*d*_t_ − *V*_b_/*d*_b_)/2 for top and bottom hBN thicknesses, *d*_t_ and *d*_b_, respectively, and ${n}_{0}=({C}_{{{{{{\rm{t}}}}}}}^{0}{V}_{{{{{{\rm{t}}}}}}}+{C}_{{{{{{\rm{b}}}}}}}^{0}{V}_{{{{{{\rm{b}}}}}}})/e$. Self-consistent solution of Eqs. (14a), (14b), and (15) includes the charge density and electric potential on each layer for a given set of applied gate voltages and thus fully incorporates the effect of screening the externally applied electric fields. Finally, to include the effect of the two polarization states, as shown in the main text for ${C}_{{{{{{\rm{t}}}}}}}^{\pm }$, we compute the capacitances separately for each case, ±, using the compressibilities, ${\nu }_{ij}^{\pm }$, calculated from the eigenstates of ${\hat{{{{{{{{\mathcal{H}}}}}}}}}}^{\pm }$, derived from the appropriate form of Eq. (4).

### Simplifications and approximations

In practice, quantum capacitances of 2D electron gases tend to dominate over geometric capacitances from external electrodes and gates due to the small energy spacing between levels. For instance, the density of states (and thus the non-interacting electronic compressibility) in monolayer graphene is of the order *ρ*_MLG_(0) ~ 10^13^ eV/cm^2^, yielding a quantum capacitance of order *C*_q_ ~ 20 fF/μm^2^. In comparison, a metallic gate separated by 10 nm of hBN from a parallel plate generates a geometric capacitance one order of magnitude smaller, *C*_g_ ~ 2 fF/μm^2^. Typical thicknesses for hBN gate dielectrics are even larger, such as the device shown in the main text with *d*_t_ ≈ 15 nm and *d*_b_ ≈ 19 nm, resulting in even smaller geometric capacitances. Additionally, in each layer of bilayer WTe_2_, screening from carriers in the nearby layer and weak interlayer coupling significantly reduces the off-diagonal compressibilities relative to the diagonal terms in Eq. (12). Together, these considerations imply a hierarchy, $| {\nu }_{11}| ,| {\nu }_{22}| \gg {C}_{{{{{{\rm{t}}}}}}}^{0},{C}_{{{{{{\rm{b}}}}}}}^{0},{C}_{i}\gg | {\nu }_{12}| ,| {\nu }_{21}|$ (ignoring factors of *e*^2^ preceding the *ν*_*ij*_ terms). As a result, the diagonal compressibilities *ν*_*ii*_ dominate the behavior of *C*_t_ and *C*_b_, enabling an approximate form for Eqs. (11a–11c),

16a $${C}_{{{{{{\rm{t}}}}}}}\approx {C}_{{{{{{\rm{t}}}}}}}^{0}\left(1-\frac{{C}_{{{{{{\rm{t}}}}}}}^{0}}{{e}^{2}{\nu }_{11}}\right),$$

16b $${C}_{{{{{{\rm{b}}}}}}}\approx {C}_{{{{{{\rm{b}}}}}}}^{0}\left(1-\frac{{C}_{{{{{{\rm{b}}}}}}}^{0}}{{e}^{2}{\nu }_{22}}\right),$$

as shown in Eq. (1) in the main text. While the numerical computations shown in the paper make use of the full Eq. (11a), deviations of the approximate formulas from the exact quantities are negligible and thus the simpler form is given in main text Eq. (1) to facilitate understanding.

### Capacitance relation to polarization

To extract the spontaneous polarization from the measured capacitance, we seek the change in the polarization,

17 $${{\Delta }}{P}_{{{{{{\rm{s}}}}}}}={P}^{+}-{P}^{-},$$

obtained by integrating the difference of *dipole compressibilities*, ∂*p*/∂*n*_0_ in each polarization state,

18 $${{\Delta }}{P}_{{{{{{\rm{s}}}}}}}={d}_{i}\int \left(\frac{\partial {p}^{+}}{\partial {n}_{0}}-\frac{\partial {p}^{-}}{\partial {n}_{0}}\right){{{{{\rm{d}}}}}}{n}_{0}+{{\Delta }}{P}_{0},$$

defined up to a constant of integration, Δ*P*_0_, which we take to be zero at large hole densities, where the density-dependent spontaneous polarization appears to saturate. The dipole compressibility in each state, ±, is defined by

19 $$\frac{\partial {p}^{\pm }}{\partial {n}_{0}}=\frac{\partial {n}_{1}^{\pm }}{\partial {n}_{0}}-\frac{\partial {n}_{2}^{\pm }}{\partial {n}_{0}}.$$

Equation (18) together with Eq. (19) is employed to compute the “Exact” spontaneous polarization in the model calculations shown in Fig. 4d. In the experiment, we do not have direct access to the partial derivatives in Eq. (19); however, the symmetry of our device allows an approximate form of Eq. (18) to be related to Δ*P*_s_.

Following ref. ^22^, we express the dipole compressibility in Eq. (19) as,

20 $$\frac{\partial p}{\partial {n}_{0}}=\frac{\partial {n}_{1}}{\partial {V}_{{{{{{\rm{t}}}}}}}}\frac{\partial {V}_{{{{{{\rm{t}}}}}}}}{\partial {n}_{0}}+\frac{\partial {n}_{1}}{\partial {V}_{{{{{{\rm{b}}}}}}}}\frac{\partial {V}_{{{{{{\rm{b}}}}}}}}{\partial {n}_{0}}-\frac{\partial {n}_{2}}{\partial {V}_{{{{{{\rm{t}}}}}}}}\frac{\partial {V}_{{{{{{\rm{t}}}}}}}}{\partial {n}_{0}}-\frac{\partial {n}_{2}}{\partial {V}_{{{{{{\rm{b}}}}}}}}\frac{\partial {V}_{{{{{{\rm{b}}}}}}}}{\partial {n}_{0}},$$

where we have dropped the label indexing the polarization state. We then employ

21 $$e\left(\begin{array}{c}\delta {n}_{1}\\ \delta {n}_{2}\end{array}\right)=[1-\hat{C}{({e}^{2}\hat{\nu }+\hat{C})}^{-1}]\left(\begin{array}{c}{C}_{{{{{{\rm{t}}}}}}}^{0}\delta {V}_{{{{{{\rm{t}}}}}}}\\ {C}_{{{{{{\rm{b}}}}}}}^{0}\delta {V}_{{{{{{\rm{b}}}}}}}\end{array}\right),$$

to obtain the partials ∂*n*_*i*_/∂*V*_t,b_ and finally rewrite the dipole compressibility in terms of the average geometric capacitance, $\langle C\rangle =({C}_{{{{{{\rm{b}}}}}}}^{0}+{C}_{{{{{{\rm{t}}}}}}}^{0})/2$, and the asymmetry, $\delta =({C}_{{{{{{\rm{b}}}}}}}^{0}-{C}_{{{{{{\rm{t}}}}}}}^{0})/({C}_{{{{{{\rm{b}}}}}}}^{0}+{C}_{{{{{{\rm{t}}}}}}}^{0})$,

22 $$\begin{array}{l}\frac{\partial p}{\partial {n}_{0}}=\frac{{C}_{i}}{{\langle C\rangle }^{2}}\left(1+\frac{\langle C\rangle }{2{C}_{i}}\right)({C}_{{{{{{\rm{b}}}}}}}-{C}_{{{{{{\rm{t}}}}}}})\\ +\frac{4{C}_{i}}{{\langle C\rangle }^{2}}\left[\frac{2\langle C\rangle -{C}_{{{{{{\rm{b}}}}}}}-{C}_{{{{{{\rm{t}}}}}}}}{4}-\left(1+\frac{\langle C\rangle }{2{C}_{i}}\right){C}_{{{{{{\rm{p}}}}}}}\right]\delta +{{{{{{{\mathcal{O}}}}}}}}({\delta }^{2}).\end{array}$$

For the device shown in the main text (Device A in Supplementary Table 1), *δ* ≈ 0.11 and 〈*C*〉/2*C*_*i*_ ≈ 0.067. Keeping only the leading order terms in these two small parameters, we obtain

23 $$\frac{\partial p}{\partial {n}_{0}}\approx -\frac{{C}_{i}}{{\langle C\rangle }^{2}}({C}_{{{{{{\rm{t}}}}}}}-{C}_{{{{{{\rm{b}}}}}}}).$$

Therefore, we have

24 $$\frac{\partial {{\Delta }}{P}_{{{{{{\rm{s}}}}}}}}{\partial {n}_{0}}	={d}_{i}\left(\frac{\partial {p}^{+}}{\partial {n}_{0}}-\frac{\partial {p}^{-}}{\partial {n}_{0}}\right)\\ 	 \approx \kern-1pt-{d}_{i}\frac{{C}_{i}}{{\langle C\rangle }^{2}}\left[({C}_{{{{{{\rm{t}}}}}}}^{+}-{C}_{{{{{{\rm{b}}}}}}}^{+})-({C}_{{{{{{\rm{t}}}}}}}^{-}-{C}_{{{{{{\rm{b}}}}}}}^{-})\right]\\ 	 =-{d}_{i}\frac{{C}_{i}}{{\langle C\rangle }^{2}}({{\Delta }}{C}_{{{{{{\rm{t}}}}}}}-{{\Delta }}{C}_{{{{{{\rm{b}}}}}}}),$$

where ${{\Delta }}{C}_{{{{{{\rm{t(b)}}}}}}}\equiv {C}_{{{{{{\rm{t(b)}}}}}}}^{+}-{C}_{{{{{{\rm{t(b)}}}}}}}^{-}$. To evaluate this further, we first rearrange Eq. (1) and explicitly show the *E*_⊥_ dependence (ignoring factors of *e*^2^),

25 $$\frac{{C}_{{{{{{\rm{t}}}}}}}({E}_{\perp })}{{\left({C}_{{{{{{\rm{t}}}}}}}^{0}\right)}^{2}}\,\approx\, \frac{1}{{C}_{{{{{{\rm{t}}}}}}}^{0}}-\frac{1}{{\nu }_{11}({E}_{\perp })},$$

which implies

26 $$\frac{{{\Delta }}{C}_{{{{{{\rm{t}}}}}}}({E}_{\perp })}{{\left({C}_{{{{{{\rm{t}}}}}}}^{0}\right)}^{2}}=\frac{{C}_{{{{{{\rm{t}}}}}}}^{+}({E}_{\perp })}{{\left({C}_{{{{{{\rm{t}}}}}}}^{0}\right)}^{2}}-\frac{{C}_{{{{{{\rm{t}}}}}}}^{-}({E}_{\perp })}{{\left({C}_{{{{{{\rm{t}}}}}}}^{0}\right)}^{2}}=\frac{1}{{\nu }_{11}^{-}({E}_{\perp })}-\frac{1}{{\nu }_{11}^{+}({E}_{\perp })}.$$

Similarly, we have

27 $$\frac{{{\Delta }}{C}_{{{{{{\rm{b}}}}}}}({E}_{\perp })}{{\left({C}_{{{{{{\rm{b}}}}}}}^{0}\right)}^{2}}=\frac{1}{{\nu }_{22}^{-}({E}_{\perp })}-\frac{1}{{\nu }_{22}^{+}({E}_{\perp })}.$$

Using the fact that switching the polarization state is equivalent to a mirror operation between the layers, we find

28 $${\nu }_{22}^{\pm }({E}_{\perp })={\nu }_{11}^{\mp }(-{E}_{\perp }),$$

a symmetry that is evident in the identity relating the Hamiltonian in the two polarization states, Eq. (6), and the subsequent compressibilities derived from each. Physically, this implies that measuring the capacitance from one side of the device in the two polarization states is related to a measurement of the capacitance from both sides of the device by geometrical factors. Thus, we invoke Eq. (28) to relate Eqs. (26) and (27),

29 $$\frac{{{\Delta }}{C}_{{{{{{\rm{b}}}}}}}({E}_{\perp })}{{\left({C}_{{{{{{\rm{b}}}}}}}^{0}\right)}^{2}}=\frac{1}{{\nu }_{11}^{+}(-{E}_{\perp })}-\frac{1}{{\nu }_{11}^{-}(-{E}_{\perp })}=-\frac{{{\Delta }}{C}_{{{{{{\rm{t}}}}}}}(-{E}_{\perp })}{{\left({C}_{{{{{{\rm{t}}}}}}}^{0}\right)}^{2}},$$

or equivalently

30 $${{\Delta }}{C}_{{{{{{\rm{b}}}}}}}({E}_{\perp })=-\frac{{\left({C}_{{{{{{\rm{b}}}}}}}^{0}\right)}^{2}}{{\left({C}_{{{{{{\rm{t}}}}}}}^{0}\right)}^{2}}{{\Delta }}{C}_{{{{{{\rm{t}}}}}}}(-{E}_{\perp })=-{\left(\frac{{d}_{{{{{{\rm{t}}}}}}}}{{d}_{{{{{{\rm{b}}}}}}}}\right)}^{2}{{\Delta }}{C}_{{{{{{\rm{t}}}}}}}(-{E}_{\perp }),$$

for hBN dielectrics on both sides of the device. Finally, we insert this identity into Eq. (24),

31 $$\frac{\partial {{\Delta }}{P}_{{{{{{\rm{s}}}}}}}({E}_{\perp })}{\partial {n}_{0}}=-{d}_{i}\frac{{C}_{i}}{{\langle C\rangle }^{2}}\left[{{\Delta }}{C}_{{{{{{\rm{t}}}}}}}({E}_{\perp })+{\left(\frac{{d}_{{{{{{\rm{t}}}}}}}}{{d}_{{{{{{\rm{b}}}}}}}}\right)}^{2}{{\Delta }}{C}_{{{{{{\rm{t}}}}}}}(-{E}_{\perp })\right],$$

solely in terms of measured Δ*C*_t_(*E*_⊥_). Integrating this expression from the largest hole density measured, *n*_l_, up to *n*_0_, we obtain

32 $$\begin{array}{rcl}{{\Delta }}{P}_{{{{{{\rm{s}}}}}}}({E}_{\perp },{n}_{0})&\approx &-{d}_{i}\frac{{C}_{i}}{{\langle C\rangle }^{2}}\displaystyle\int_{{n}_{{{{{{\rm{l}}}}}}}}^{{n}_{0}}\,\bigg[{{\Delta }}{C}_{{{{{{\rm{t}}}}}}}({E}_{\perp },{n}_{0}^{\prime})\\ &&+\left.{\left(\frac{{d}_{{{{{{\rm{t}}}}}}}}{{d}_{{{{{{\rm{b}}}}}}}}\right)}^{2}{{\Delta }}{C}_{{{{{{\rm{t}}}}}}}(-{E}_{\perp },{n}_{0}^{\prime})\right]\ {{{{{{{\rm{d}}}}}}}}{n}_{0}^{\prime}.\end{array}$$

Evaluating at *E*_⊥_ = 0, we arrive at the approximate integral used to determine the measured polarization in Fig. 4b,

33 $${{\Delta }}{P}_{{{{{{\rm{s}}}}}}}(0,{n}_{0})\approx -{d}_{i}\frac{{C}_{i}}{{\langle C\rangle }^{2}}\left[1+{\left(\frac{{d}_{{{{{{\rm{t}}}}}}}}{{d}_{{{{{{\rm{b}}}}}}}}\right)}^{2}\right]\int\nolimits_{{n}_{{{{{{\rm{l}}}}}}}}^{{n}_{0}}\,{{\Delta }}{C}_{{{{{{\rm{t}}}}}}}(0,{n}_{0}^{\prime})\ {{{{{{{\rm{d}}}}}}}}{n}_{0}^{\prime}.$$

The same expression is employed in Fig. 4d to calculate the “Approx.” curve based on computed capacitance data, illustrating the small deviation from the exact polarization introduced by making the approximations outlined in this section. A small constant of integration is included in the total measured spontaneous polarization shown in Fig. 4b, *P*_0_ = 0.42 e/cm, inferred by matching the magnitude of the measured Δ*P*_s_(*n*_0_) with the calculated curve. The latter curve exhibits saturation of Δ*P*_s_ at large hole densities (taken to be zero) and thus offers a lower bound on the constant of integration and ultimately the maximum value of the polarization near charge neutrality.

### First-principles calculations

As the essential ingredient to interpret the capacitance in experiments, the low-energy model in Eq. (4) is well supported by first-principles calculations. The most essential element for the capacitance is the polarization or the occupation difference between the top and bottom layer. In Supplementary Fig. 6, we show such layer character in the band structure near one group of valleys from the first-principles calculations compared with those from the low-energy model. By tracing the valence band, we find that bands from the low-energy model and first-principles calculation show similar tilting, together with similar layer-occupancy variation. This striking similarity demonstrates that the low-energy model is indeed capable of capturing the essential physics for the capacitance calculation.

The supporting density functional theory (DFT) calculations shown in Supplementary Fig. 6b are performed with the Vienna Ab initio Simulation Package using the PBEsol functional. The plane wave basis cutoff is 300 eV. The atomic structures are relaxed until forces on every atom are <0.01 eV/Å and the vacuum layer is >15 Å. The vdW functional used here is the zero damping DFT-D3 method with small modifications: the vdW correction is only applied to atomic pairs from different layers, since monolayers can be calculated well without vdW correction. This modification ensures that the structure of the sub-layer of the bilayer will not be affected by an inappropriate vdW correction, leading to the same lattice constant for the monolayer and bilayer system.

## Supplementary information

Supplementary Information

Peer Review File

## Data Availability

All relevant data in this study are available from the corresponding authors upon reasonable request.

## Code availability

Custom computer codes used to support the conclusions in this study are available from the corresponding authors upon reasonable request.

## References

[1] Anderson PW, Blount EI (1965). Symmetry considerations on martensitic transformations: “ferroelectric” metals?. Phys. Rev. Lett..

[2] Rabe, K. M., Dawber, M., Lichtensteiger, C., Ahn, C. H., & Triscone, J. M. In *Physics of Ferroelectrics*, (eds Rabe, K. M., Ahn, C. H. & Triscone, J.-M.) Vol. 105, 1–30 (Springer, 2007).

[3] Shi Y (2013). A ferroelectric-like structural transition in a metal. Nat. Mater..

[4] Liebmann M (2016). Giant Rashba-type spin splitting in ferroelectric GeTe(111). Adv. Mater..

[5] Kim TH (2016). Polar metals by geometric design. Nature.

[6] Filippetti A, Fiorentini V, Ricci F, Delugas P, Íñiguez J (2016). Prediction of a native ferroelectric metal. Nat. Commun..

[7] Benedek NA, Birol T (2016). ‘Ferroelectric’ metals reexamined: fundamental mechanisms and design considerations for new materials. J. Mater. Chem. C.

[8] Rischau CW (2017). A ferroelectric quantum phase transition inside the superconducting dome of Sr_1−*x*_Ca_*x*_TiO_3−*δ*_. Nat. Phys..

[9] Yuan S (2019). Room-temperature ferroelectricity in MoTe_2_ down to the atomic monolayer limit. Nat. Commun..

[10] Fei Z (2018). Ferroelectric switching of a two-dimensional metal. Nature.

[11] Sharma P (2019). A room-temperature ferroelectric semimetal. Sci. Adv..

[12] Xiao J (2020). Berry curvature memory through electrically driven stacking transitions. Nat. Phys..

[13] Wu S (2018). Observation of the quantum spin Hall effect up to 100 kelvin in a monolayer crystal. Science.

[14] Fatemi V (2018). Electrically tunable low-density superconductivity in a monolayer topological insulator. Science.

[15] Sajadi E (2018). Gate-induced superconductivity in a monolayer topological insulator. Science.

[16] Xu SY (2018). Electrically switchable Berry curvature dipole in the monolayer topological insulator WTe_2_. Nat. Phys..

[17] Ma Q (2019). Observation of the nonlinear Hall effect under time-reversal-symmetric conditions. Nature.

[18] Yang Q, Wu M, Li J (2018). Origin of two-dimensional vertical ferroelectricity in WTe_2_ bilayer and multilayer. J. Phys. Chem. Lett..

[19] Liu X (2019). Vertical ferroelectric switching by in-plane sliding of two-dimensional bilayer WTe_2_. Nanoscale.

[20] Wang L (2016). Direct observation of a long-range field effect from gate tuning of nonlocal conductivity. Phys. Rev. Lett..

[21] Young AF, Levitov LS (2011). Capacitance of graphene bilayer as a probe of layer-specific properties. Phys. Rev. B.

[22] Hunt, B. M. et al. Direct measurement of discrete valley and orbital quantum numbers in bilayer graphene. *Nat. Commun*. **8**, 948 (2017). 10.1038/s41467-017-00824-w PMC5715057 29038518

[23] Telford EJ (2018). Via method for lithography free contact and preservation of 2D materials. Nano Lett..

[24] Fei Z (2017). Edge conduction in monolayer WTe_2_. Nat. Phys..

[25] Young AF (2012). Electronic compressibility of layer-polarized bilayer graphene. Phys. Rev. B.

[26] Du ZZ, Wang CM, Lu H-Z, Xie XC (2018). Band signatures for strong nonlinear Hall effect in bilayer wte_2_. Phys. Rev. Lett..

[27] Li L, Wu M (2017). Binary compound bilayer and multilayer with vertical polarizations: two-dimensional ferroelectrics, multiferroics, and nanogenerators. ACS Nano.

[28] Zheng Z (2020). Unconventional ferroelectricity in Moiré heterostructures. Nature.

[29] Yasuda K, Wang X, Watanabe K, Taniguchi T, Jarillo-Herrero P (2021). Stacking-engineered ferroelectricity in bilayer boron nitride. Science.

[30] Vizner Stern M (2021). Interfacial ferroelectricity by van der Waals sliding. Science.

[31] Woods CR (2021). Charge-polarized interfacial superlattices in marginally twisted hexagonal boron nitride. Nat. Commun..

[32] Zhang Y, Liu T, Fu L (2021). Electronic structures, charge transfer, and charge order in twisted transition metal dichalcogenide bilayers. Phys. Rev. B.

[33] Wang, Y. et al. Tunable ferroelectricity in hBN intercalated twisted double-layer graphene. Preprint at https://arxiv.org/abs/2102.12398 (2021).

[34] Muechler L, Alexandradinata A, Neupert T, Car R (2016). Topological nonsymmorphic metals from band inversion. Phys. Rev. X.

