# Supplementary material for: Direct measurement of ferroelectric polarization in a tunable semimetal
Source: Nat Commun. 2021 Sep 6;12:5298. doi: 10.1038/s41467-021-25587-3 (PMC8421369; doi:10.1038/s41467-021-25587-3)
Supplement: Supplementary file 1 — Supplementary Information [file 41467_2021_25587_MOESM1_ESM.pdf]

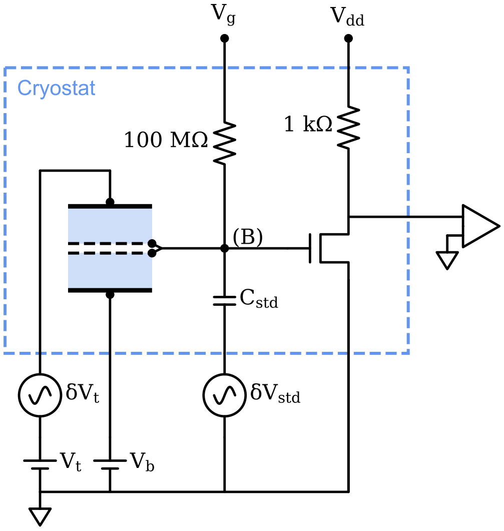

Supplementary Fig. 1. **Diagram of the capacitance bridge circuit.** The capacitance bridge and amplifier circuit is mounted next to the device in the cryostat sample space (dashed box). The circuit is supplied with external dc gate voltages V_t, V_b and ac excitation voltages applied to the top gate δV_t and the standard capacitor δV_{std}. The deviation from null signal at the bridge balance point (B) is amplified by a high-electron-mobility transistor (HEMT) held at optimal gain by a dc gate voltage V_g and drain current set by V_{dd}.

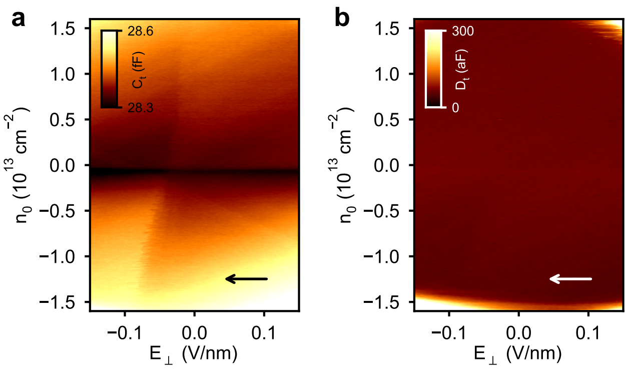

Supplementary Fig. 2. **Capacitance and dissipation in the compressibility regime. a** Capacitance (in-phase) data from a single sweep direction (see arrow) from the same measurement shown in Fig. **2**. **b** Dissipation (out-of-phase) signal from the same measurement, showing no features in the relevant parameter space. Here the dissipation is offset by 1.23 fF to shift the average value of the dissipation to zero. The scale of **b** is set to match **a** for comparison. The origin of the shifted mean value of the dissipation is systematic phase shifts in the combined capacitance bridge and cryostat wiring. The bright features in the corners of the scan range in the dissipation signal arise from deviations in the cryogenic amplifier operating point, and do not affect the analyses described in the main text.

Device	A (μm^2)	d_b (nm)	d_t (nm)	Bottom gate	Top gate
A	16.3	19	15	Gr	Au
B	40.6	41	13	Pt	Au
C	23.7	32	40	Gr	Au
D	22.0	20	30	Gr	Au

Supplementary Table 1. Table of device parameters, including area (A), bottom and top gate dielectric thicknesses (d_b and d_t), and bottom and top gate materials (Gr = graphite). All data shown in the main text were obtained from Device A.

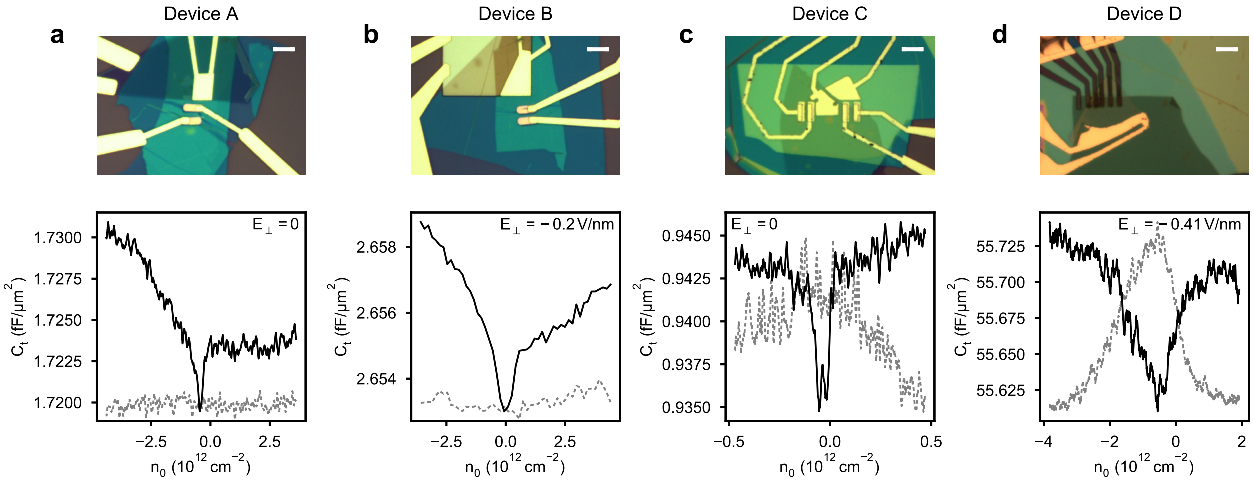

Supplementary Fig. 3. **Additional devices in the study.** **a-d** Optical images (top) and measured capacitance at fixed electric field for Devices A-D, respectively. Gray dashed curves show the dissipation signal, vertically shifted up to the minimum value of the capacitance for comparison. All scale bars are 5 µm.

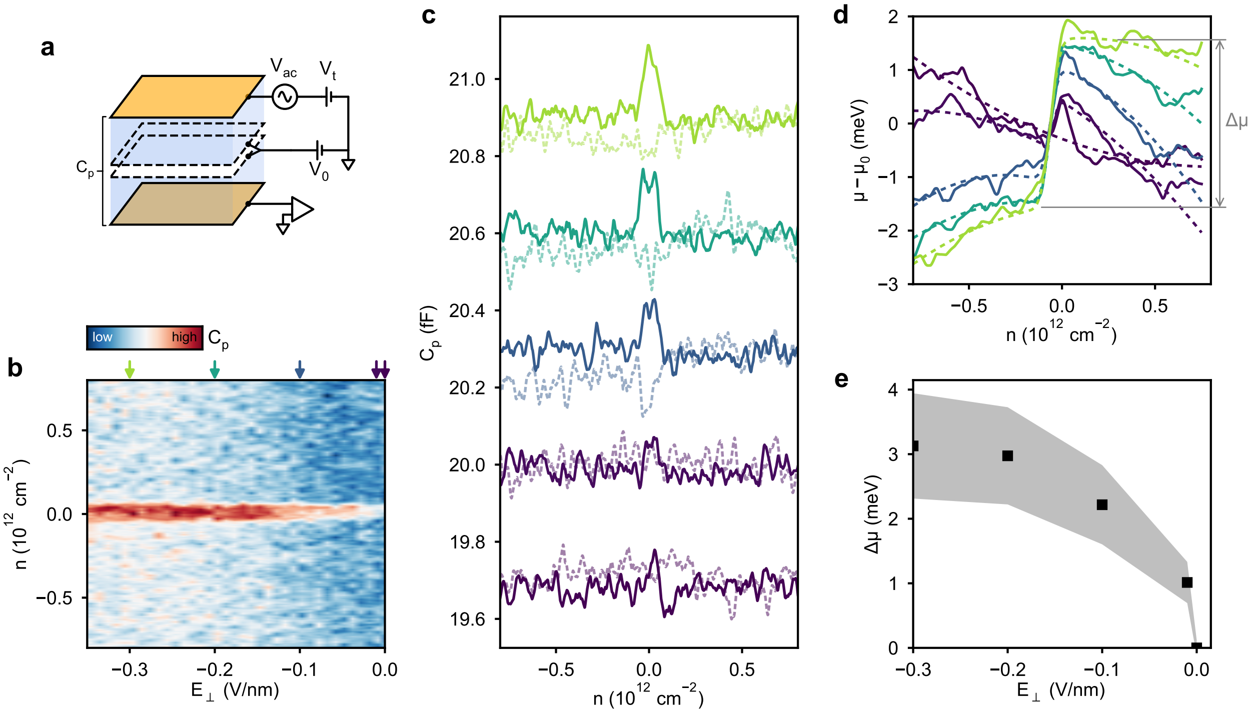

Supplementary Fig. 4. **Determination of the electric-field-induced gap.** **a** Schematic of the penetration field capacitance C_p circuit, measuring the capacitance between the top and bottom gate. **b** Measured C_p dependence on electric field $E_\perp$ and density n, showing an incompressible (large C_p) feature at charge neutrality (measured at 4 K). **c** Selected C_p traces from **b** as a function of density at fixed $E_\perp$ (indicated by colored arrows at the top of **b**), with dashed lines showing the dissipation (out-of-phase) signal for each trace on the same scale. Curves shifted vertically for clarity. **d** Shift of the chemical potential $\mu - \mu_0 = (e/\langle C\rangle^2)\int C_p\, dn$ calculated by integrating the traces in **c** with respect to density, using average geometric capacitance $\langle C\rangle = (C_t^0 + C_b^0)/2$ and setting the constant of integration μ_0 to the mid-gap value of the chemical potential. Dashed lines indicate error-function fits used to extract the jump the in chemical potential $\Delta\mu$ at charge neutrality. **e** Jump in the chemical potential at charge neutrality extracted from fits in **d**, a quantitative measure of the electric-induced gap as a function of $E_\perp$. Shaded region indicates the total uncertainty of the extracted values including fitting errors as well as uncertainty in the value of the reference capacitor, $C_{\rm std} = 13 \pm 3$ pF.

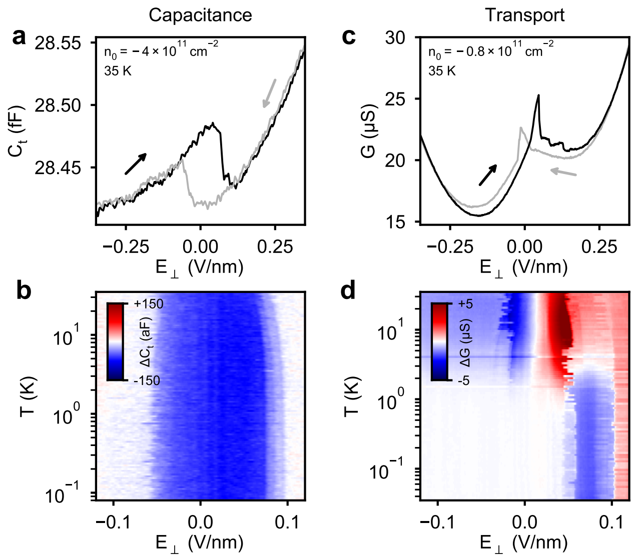

Supplementary Fig. 5. **Temperature dependence of the hysteresis. a** Capacitance hysteresis loop from Device A at the highest temperature measured, 35 K. **b** Temperature dependence of the hysteretic difference ΔC_t (described in the main text), showing little change in the capacitance signal and critical fields, $E_c^{\pm}$. **c** Two-terminal conductance from Device C showing switching behavior at a comparable low hole density to **a**. **d** Temperature dependence of the difference of conductance sweeps, ΔG, showing a weakening signature below $\sim$1 K. In contrast, capacitance measurements suggest that the polarization is not strongly affected by temperature in this range.

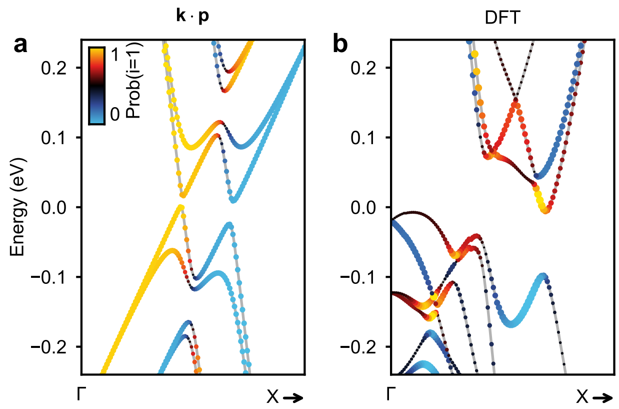

Supplementary Fig. 6. **Calculated bands with layer polarization. a** Calculated bands from the low-energy $\mathbf{k}\cdot\mathbf{p}$ model (for $E_\perp = 0$) and **b** density functional theory (DFT). Color and symbol size for each eigenstate denotes the normalized probability of appearing in layer 1, Prob$(i=1)$, according to the color scale in **a**. The probability of appearing in layer 2 is complementary, Prob$(i=2) = 1 - $Prob$(i=1)$.

$\phi_1^0 = -0.08\,\text{eV}$	$\phi_2^0 = 0.03\,\text{eV}$
$q_1 = 0.1\pi$	$q_2 = 0.15\pi$
$\lambda_x = 0.2535\,\text{eV Å}$	$\lambda_y = 0$
$t = 2.5355\,\text{eV Å}$	$m = 0.1\,\text{eV}$
$\gamma = 0.05\,\text{eV}$	$\eta_1 = -\eta_2 = -1$
$v = 3.38\,\text{eV Å}$	$\alpha = 2.857\,\text{eV Å}^2$

Supplementary Table 2. Parameters employed in $\mathbf{k}\cdot\mathbf{p}$ calculation, following Eqs. 3–5. These parameters are obtained by fitting the polarization from the low-energy model to Fig. **4a**, and hence are different from those in Ref. 26.

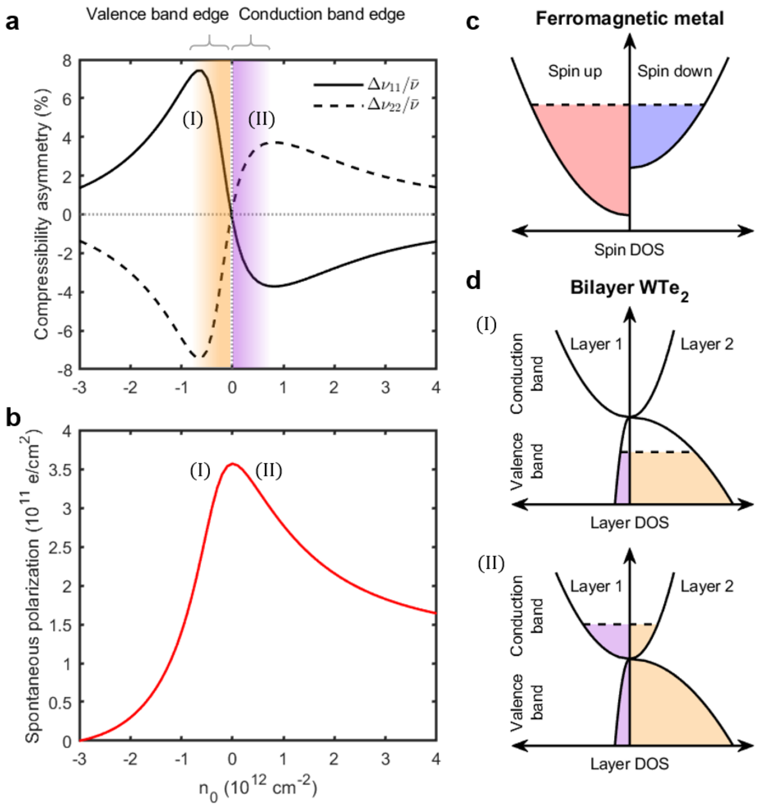

Supplementary Fig. 7. **Electron-hole asymmetry in the polarization. a** Asymmetry of the layer-specific compressibilities ν_{ii} for layers $i = 1, 2$ determined by the deviation from the average, $\Delta\nu_{ii}/\bar{\nu} = (\nu_{ii} - \bar{\nu})/\bar{\nu}$ with $\bar{\nu} = (\nu_{11} + \nu_{22})/2$. Compressibilities were evaluated at $E_\perp = 0$ for one polarization state using the $\mathbf{k} \cdot \mathbf{p}$ model. The increased asymmetry of the layer compressibilities in the valence band relative to the conduction band leads to a larger dipole compressibility $\partial p/\partial n_0$ in the former. That is, as electrons fill the valence band, macroscopic polarization accumulates, reaching a maximum at charge neutrality. The dipole compressibility then switches sign and dipoles with opposite polarization begin filling the conduction band, partially screening the polarization from the valence band. **b** Calculated spontaneous polarization, as described in the main text, arises from the integrated difference between the dipole compressibilities in the two polarization states. **c** Schematic spin density of states (DOS) in a ferromagnetic metal showing an imbalance in the DOS for one spin population relative to the other. **d** An analogous schematic/model for the layer DOS in bilayer WTe_2 based on the trend of the layer-specific compressibilities. An imbalance in the DOS (or compressibility) in each layer leads to an accumulation of dipoles and spontaneous polarization. Electron-hole asymmetry in the band structure leads to a total polarization that depends on filling, with (I) filled valence band states producing a larger layer imbalance relative to (II) conduction band states.

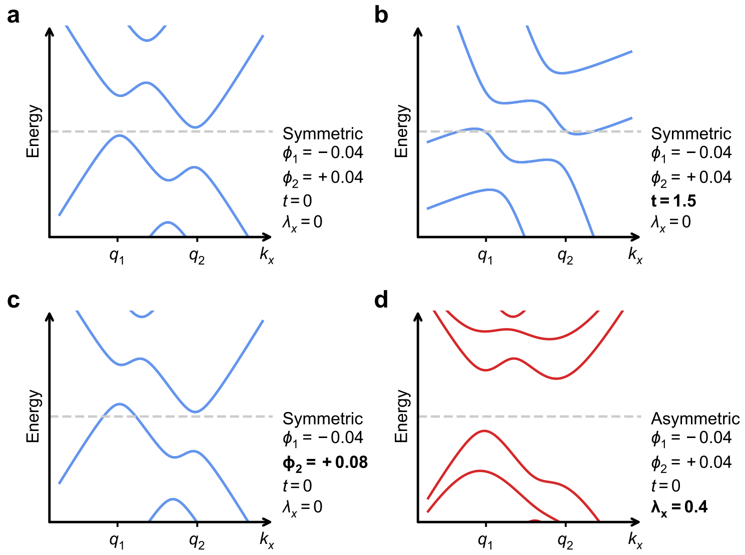

Supplementary Fig. 8. **Origin of e–h asymmetry in the $\mathbf{k} \cdot \mathbf{p}$ bands.** **a** Calculated $\mathbf{k} \cdot \mathbf{p}$ bands without tilting t or spin-orbit coupling (SOC) λ_x, with $\phi_1 = -\phi_2$. The bands are symmetric around charge neutrality (denoted by dashed line) upon interchanging valleys ($q_1 \leftrightarrow q_2$). **b** Including tilting preserves this e–h symmetry. **c** With asymmetric on-site potentials, $\phi_1 \neq -\phi_2$, the chemical potential is shifted from the charge neutral point, but e–h symmetry remains. **d** Finite SOC generally opens a spin gap in each valley. Due to the absence of in-plane mirror symmetry and mirror-z symmetry in the system (inequivalent layers), the effect of SOC is different in each layer and the spin gap differs in the two valleys. SOC thus lifts the e–h symmetry of the layer-polarized bands and leads to asymmetry in the polarization. All parameter values are given in eV and enlarged to highlight the effect on the bands.

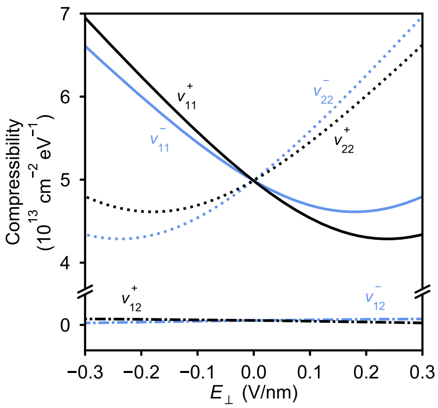

Supplementary Fig. 9. **Complete compressibility matrix for the bilayer system.** Comparison of compressibility magnitudes highlighting the secondary role of differential interlayer couplings $\nu_{12} = \nu_{21} = dn_1/d\phi_2 = dn_2/d\phi_1$.
